# DJ-1 Acts as a Scavenger of α-Synuclein Oligomers and Restores Monomeric Glycated α-Synuclein

**DOI:** 10.3390/biom11101466

**Published:** 2021-10-06

**Authors:** Tamr B. Atieh, Jonathan Roth, Xue Yang, Cody L. Hoop, Jean Baum

**Affiliations:** Department of Chemistry and Chemical Biology, Rutgers University, Piscataway, NJ 08854, USA; tamr@chem.rutgers.edu (T.B.A.); jr1214@chem.rutgers.edu (J.R.); yangxuechemistry@hotmail.com (X.Y.); cody.hoop@rutgers.edu (C.L.H.)

**Keywords:** α-synuclein, glycation, DJ-1, Parkinson’s disease, protein–protein interactions, nuclear magnetic resonance spectroscopy, atomic force microscopy

## Abstract

Glycation of α-synuclein (αSyn), as occurs with aging, has been linked to the progression of Parkinson’s disease (PD) through the promotion of advanced glycation end-products and the formation of toxic oligomers that cannot be properly cleared from neurons. DJ-1, an antioxidative protein that plays a critical role in PD pathology, has been proposed to repair glycation in proteins, yet a mechanism has not been elucidated. In this study, we integrate solution nuclear magnetic resonance (NMR) spectroscopy and liquid atomic force microscopy (AFM) techniques to characterize glycated N-terminally acetylated-αSyn (glyc-ac-αSyn) and its interaction with DJ-1. Glycation of ac-αSyn by methylglyoxal increases oligomer formation, as visualized by AFM in solution, resulting in decreased dynamics of the monomer amide backbone around the Lys residues, as measured using NMR. Upon addition of DJ-1, this NMR signature of glyc-ac-αSyn monomers reverts to a native ac-αSyn-like character. This phenomenon is reversible upon removal of DJ-1 from the solution. Using relaxation-based NMR, we have identified the binding site on DJ-1 for glycated and native ac-αSyn as the catalytic pocket and established that the oxidation state of the catalytic cysteine is imperative for binding. Based on our results, we propose a novel mechanism by which DJ-1 scavenges glyc-ac-αSyn oligomers without chemical deglycation, suppresses glyc-ac-αSyn monomer–oligomer interactions, and releases free glyc-ac-αSyn monomers in solution. The interference of DJ-1 with ac-αSyn oligomers may promote free ac-αSyn monomer in solution and suppress the propagation of toxic oligomer and fibril species. These results expand the understanding of the role of DJ-1 in PD pathology by acting as a scavenger for aggregated αSyn.

## 1. Introduction

As the population shifts towards an aging society, it is imperative to understand the effect of aging on neurodegenerative diseases. One result of aging is the body’s inability to mitigate the harmful impacts of glucose metabolism, which produces reactive oxygen species (ROS) and reactive aldehyde species [1,2]. A direct consequence of this aldehyde formation is the non-enzymatic chemical ligation of sugar aldehydes to the side chains of susceptible proteins in the formation of advanced glycation end-products (AGEs) [3]. Protein glycation has been linked to multiple degenerative diseases such as Parkinson’s disease (PD) [4], Alzheimer’s disease [5], Huntington’s disease [6], diabetes [7], and atherosclerosis [8]. Glycation of amyloidogenic proteins associated with these diseases has been shown to induce their formation of toxic oligomers that are unable to be cleared by the cell [9,10]. A build-up of glycated protein in neurons adds another challenge against combating debilitating neurodegenerative diseases. Suppressing the aggregation of these toxic glycated species may be a viable approach toward alleviating the effects of aging-related neurodegeneration. 

Aggregation of the intrinsically disordered protein α-synuclein (αSyn) is associated with multiple neurodegenerative diseases including PD, multiple system atrophy, and Lewy Body Dementia [11,12] and leads to the formation of Lewy bodies in the substantia nigra of neurons. The misfolding and aggregation of αSyn is very complex and involves a self-association of monomers, the development of heterogenous oligomeric species that vary in size and toxicity, and progression into fibrils that are incorporated into Lewy bodies [13,14]. Although the mechanism and culprit species for PD pathogenicity has not been elucidated, ample research has highlighted the toxicity of αSyn oligomers. αSyn oligomers have been observed at elevated levels in the brains of PD patients and transgenic mouse and Drosophila models associated with disease [15,16,17]. In vivo and in vitro studies have shown that αSyn oligomers formed in multiple conditions are toxic to neurons [18,19,20,21]. Many of these oligomers are able to propagate amyloid formation [22]. These smaller oligomeric species and fragmented fibrils are able to spread cell-to-cell and seed amyloid formation in a prion-like manner in ways long, mature amyloid fibrils cannot [18]. Therefore, αSyn oligomers may serve as an early interventional target against amyloid propagation.

Glycation of αSyn, a result of aging, leads to the formation of insoluble protein plaques and toxic oligomers that do not form fibrils and are unable to be cleared by the cell and may cause neuronal death [10,23,24,25,26]. AGEs, including glycated-αSyn (glyc-αSyn), are found at significantly higher levels in neurons of PD patients than in healthy neurons [4,10,27]. A potent glycating agent in cells is methylglyoxal (MGO), which is produced as a byproduct of glucose metabolism [28]. MGO glycates lysine, arginine, and cysteine side chains of proteins and has been shown to glycate specific lysines in the N-terminus of αSyn [10,29,30]. MGO-mediated glyc-αSyn has been postulated to play a role in Lewy Body disease and can co-aggregate with native αSyn to suppress amyloid formation [31,32]. Compounding this issue, glyc-αSyn is unable to undergo proper degradation via normal cellular pathways such as ubiquitination, cellular autophagy, or synaptic transmission [10]. Gaining a molecular understanding of ways to interfere with an accumulation of these toxic oligomeric species of glyc-αSyn is a crucial step for relieving aging effects in PD patients. Therapeutics targeting glycation pathways or that chaperone harmful aggregates may enhance the neuroprotective pathways within the cell.

Central to mitigation of the adverse effects of aging glucose metabolism lies the antioxidative protein DJ-1 [33]. DJ-1 exists as a homodimeric protein in solution and is crucial for neuronal protection against oxidative stress [34,35,36]. The oxidation state of the highly conserved cysteine at position 106, a residue within the catalytic triad, is imperative to the functionality of DJ-1 [37,38]. DJ-1 can be translocated to the mitochondria to scavenge for ROS produced from glucose metabolism within the cell [39], lessening oxidative stress [40]. DJ-1 has been suggested to act as a deglycase, which may mitigate the effects of glucose metabolism [41]. Recent in vitro studies show that DJ-1 can deglycate chemically glycated lysine, arginine, and cysteine amino acids as well as repair glutathione following an MGO attack [42]. However, DJ-1′s repair mechanism for larger proteins remains inconclusive [43,44]. 

DJ-1 has been linked to early onset PD [45,46]. The DJ-1 familial mutant L166P causes reduced stability of the homodimer and leads to early onset PD [47]. DJ-1 and αSyn are thought to colocalize within neurons and may directly interact with one another to modulate αSyn aggregation kinetics [48,49,50]. Indeed, DJ-1, with Cys106 in the sulfinic acid form, has already been shown to act as a chaperone for native αSyn and inhibit protofibril and amyloid formation in vivo and in vitro [51,52], and we show further data to support these findings (Figure S1). More recently, Kumar et al. concluded that DJ-1 directly interacts with and remodels mature αSyn fibrils and produces species that are more toxic to SH-SY5Y cells than fibrils themselves [53]. In addition, DJ-1 deficiency can lead to an accumulation of αSyn in neurons, while DJ-1 overexpression leads to a decrease in αSyn levels [54]. Several attempts to ascertain the direct interactions between αSyn and DJ-1 have been unsuccessful [55,56]. DJ-1 does not exhibit tight binding to αSyn but may have transient binding as seen through bimolecular fluorescence complementation and coimmunoprecipitation [49]. Given their co-localization, it has been speculated that DJ-1 may deglycate glyc-αSyn to repair toxic formation of AGEs in neurons [57]. However, no direct evidence has been presented. A molecular view of the impact of DJ-1 on αSyn glycation in aging PD patients would aid in the design of therapeutics against detrimental effects of αSyn glycation. 

Here, through the integration of solution nuclear magnetic resonance (NMR) spectroscopy and atomic force microscopy (AFM) in solution, we characterize glyc-αSyn and its interactions with DJ-1. Throughout our study, we use N-terminally acetylated αSyn (ac-αSyn), the ubiquitous post-translational modification found in LBs, which represents the physiologically relevant form of αSyn [58,59]. N-terminal acetylation affects the conformational ensemble of the monomer and the aggregation kinetics [60,61]. Therefore, this modification is significant in its molecular interactions with other αSyn molecules or other proteins. Relaxation-based NMR illuminates the unique transient glyc-ac-αSyn monomer–oligomer binding events and suggests that the presence of DJ-1 reduces these interactions, which are recovered upon removal of DJ-1. We determine that DJ-1 interacts with glycated and native ac-αSyn through the catalytic triad and establish that the oxidation state of the catalytic cysteine is imperative for binding. Supported by AFM imaging in solution, we propose a mechanism by which DJ-1 interacts with glyc-ac-αSyn oligomers, preventing their interaction with glyc-ac-αSyn monomers, leaving a higher population of free monomers in solution. Within PD pathology, DJ-1′s function in chaperoning αSyn may prevent the rapid accumulation of aggregated αSyn within the cell, which may enable proper clearance mechanisms from the cell and reduce the effects of neurodegeneration. Therapeutics targeting the effects of glycation in conjunction with maintaining proper DJ-1 function may successfully mitigate neurodegeneration and diminish the symptoms of PD.

## 2. Materials and Methods

### 2.1. Protein Expression and Purification of Acetylated α-Synuclein

All α-synuclein, including glycated α-synuclein, used in this work is N-terminally acetylated. Acetylated α-synuclein was expressed and purified as previously described [61]. To acetylate, αSyn and NATB plasmids were co-transformed into BL21(DE3) *E. coli* cells. Cell cultures were grown in either nutrient-rich Luria Broth (LB) or minimal M9 media supplemented with ^15^N-ammonium chloride and/or ^13^C-glucose for isotopic enrichment for NMR. Cell cultures (1 L) were grown at 37 °C with shaking until they reached an OD_600_ of 0.6–0.8, at which point 1 mM IPTG was added to induce expression and incubated at 37 °C with shaking for 4 h. The cell cultures were then spun down at 4.5k rpm for 30 min and the pellet was resuspended in 20 mL of phosphate buffered saline (PBS), pH 7.4, and then homogenized three times at 10–15 k psi. The cell lysates were spun down at 20 k rpm for 30 min, and 10 mg/mL of streptomycin sulfate was added to the supernatant and mixed for 15 min at 4 °C. Once completed, the suspension was spun down again at 20 k rpm for 30 min. The supernatant was collected, mixed with 0.361 g/mL of ammonium sulfate, and incubated at 4 °C for 1 h to precipitate the proteins. The mixture was again spun down for 30 min at 20 k rpm and the supernatant was discarded. The protein pellet was dissolved in 15 mL of PBS buffer and double boiled for 20 min and allowed to cool to room temperature. The supernatant was collected after centrifugation and dialyzed against 15 mM Tris buffer overnight at 4 °C. The protein solution was filtered through a 0.22-micrometer filter and passed through an anion exchange column (Hitrap Q HP, GE Lifesciences, Piscataway, NJ, USA). Ac-αSyn was eluted with a 250 mM NaCl gradient. All protein-containing fractions (as assessed by UV_280_) were collected and dialyzed with four buffer changes against 15 mM ammonium bicarbonate and lyophilized. Protein purity was assessed via SDS-PAGE and electrospray ionization mass spectrometry (ESI-MS) to ensure proper acetylation. Lyophilized ac-αSyn powder was stored at −20 °C.

### 2.2. Expression and Purification of DJ-1

A plasmid encoding human DJ-1 with a C-terminal His-tag was purchased from Addgene (#51488). The plasmid was transformed into BL21(DE3) *E. coli* cells. Cell cultures were grown in LB or M9 media (with isotopic enrichment for NMR) at 37 °C with shaking and allowed to reach an OD_600_ of 0.6–0.8. Expression was induced with 1 mM IPTG at 20 °C overnight. Cells were harvested and resuspended in 50 mM sodium phosphate buffer (pH 8), 300 mM NaCl, 20 mM imidazole, 1 mM DTT, and 5% glycerol. These cells were homogenized three times at 10–15 k psi and centrifuged at 20 k rpm for 30 min to remove any cellular debris. The cell lysate was filtered through a 0.22-micron filter and passed over a His trap column equilibrated with 50 mM sodium phosphate buffer (pH 8), 300 mM NaCl, 20 mM imidazole, and 5% glycerol. DJ-1 was eluted from the column with 200 mM imidazole. DJ-1 fractions were collected and dialyzed against PBS overnight at 4 °C. For preparations for size exclusion chromatography (SEC), DJ-1 was concentrated using a 3 kD filter and filtered through a 0.22-micron filter. The sample was then passed over a Superdex 200 Increase 10/300 GL SEC column that was equilibrated with PBS. DJ-1 fractions were collected and promptly oxidized. DJ-1 purity was assessed via ESI-MS and SDS PAGE-gel. The C-terminal his-tag was retained. 

### 2.3. Oxidation of DJ-1

DJ-1 at 400 μM dimer concentration was incubated with 800 µM H_2_O_2_ at 4 °C overnight, and then buffer exchanged to PBS at pH 7.4 to remove any excess H_2_O_2_. The concentration of dimeric DJ-1 was measured using A_280_ with a molar extinction coefficient of 8400 M^−1^cm^−1^ (4200 M^−1^cm^−1^ monomer). Unless otherwise stated, DJ-1 is oxidized to the sulfinic acid form before experiments, as confirmed using ESI-MS.

### 2.4. Glycation of Acetylated α-Synuclein

Lyophilized ac-αSyn was dissolved in PBS at pH 7.4 and subsequently passed through a 100 kD filter to remove aggregates and diluted to a final concentration of 50 μM monomeric ac-αSyn in PBS. The protein sample was incubated with 50 mM MGO at 37 °C for 24 h. All MGO was removed via dialysis with four buffer exchanges with PBS. To prepare samples for experiments, glycated ac-αSyn (glyc-ac-αSyn) was passed through a 100 kD filter, washed with PBS, and concentrated with a 3 kD filter. Protein concentrations of monomeric glyc-ac-αSyn were assessed via a BCA assay.

### 2.5. Reaction Conditions for DJ-1 and Glycated ac-α-Synuclein

For experiments on glyc-ac-αSyn in the presence of DJ-1 (+DJ-1), 100 μM glyc-ac-αSyn was incubated with 20 μM DJ-1 for 1 h at 37 °C in PBS at pH 7.4. For experiments on glyc-ac-αSyn after the removal of DJ-1 (–DJ-1), DJ-1 was removed by passing the glyc-ac-αSyn+DJ-1 solution over a His-trap to remove the His-tagged DJ-1. The glyc-ac-αSyn incubated with DJ-1 was assessed for purity by SDS-PAGE gel to ensure proper removal of DJ-1, and final concentration of glyc-ac-αSyn was determined using a BCA assay.

### 2.6. NMR ^1^H–^15^N 2D Correlation Spectra and ^15^N-R_2_ Experiments

NMR experiments were performed on 250 μM uniformly ^15^N-labelled native or glycated ac-αSyn at 15 °C. Lyophilized native or glycated ac-αSyn powder was dissolved in PBS buffer, pH 7.4 and filtered through a 100 kD centrifugal filter to remove large aggregates. Protein was concentrated with a 3 kD centrifugal filter and concentrations were measured via a BCA assay. NMR experiments on uniformly ^15^N-labelled DJ-1 (500 μM monomer equivalent) in PBS buffer, pH 7.4 were performed at 25 °C. All experiments were performed at 700 MHz ^1^H Larmor frequency.

For ac-αSyn samples, ^15^N-transverse relaxation rates (R_2_) were measured from a series of heteronuclear single quantum coherence (HSQC)-based 2D ^1^H–^15^N correlation spectra implementing the Carr-Purcell-Meiboom-Gill (CPMG) pulse sequence with varying relaxation delays: 8, 16, 32, 64, 72, 128, 160, 192, 256, 288, 320, 352, and 384 ms. ^15^N-R_2_ rates of ^15^N-DJ-1 were measured from a series of transverse-relaxation optimized spectroscopy (TROSY) ^1^H–^15^N correlation spectra using the CPMG pulse sequence with varying relaxation delays: 0, 8, 16, 24, 32, 40, 48, 56, 64, 72, 80 ms.

All titrations involve acquiring a ^1^H–^15^N 2D NMR spectrum of the ^15^N-labelled protein followed by addition of the natural abundance protein. After each subsequent protein addition, the sample was incubated at 37 °C for one hour to help facilitate the DJ-1–ac-αSyn reaction before acquisition.

^1^H–^15^N-HSQC, ^1^H–^15^N-TROSY, and ^15^N-R_2_ experiments were processed via NMRPipe [62] and analyzed in Sparky [63] software. ^15^N-R_2_ rates were measured by fitting a single exponential decay function to the peak intensities of the decay curves using the relaxation peak heights (rh) program in Sparky. 

### 2.7. Thioflavin T Assay

Lyophilized native or glycated ac-αSyn was dissolved in PBS, passed through a 100 kD filter to remove large aggregates, and concentrated and washed using a 3 kD centrifugal filter. Thioflavin T (ThT) reactions consisted of 70 μM native or glycated ac-αSyn with 20 μM ThT in PBS. Where indicated, DJ-1 was added to samples at 140 μM. A total of 100 μL of the samples were aliquoted into clear-bottom 96-well plate with one Teflon bead to each reaction, sealed with Axygen sealing tape (Corning), and shaken at 600 rpm at 37 °C in a POLARstar Omega fluorimeter (BMG Labtech) for over 100 h. Fluorescence was monitored every 33 min. 

### 2.8. Thioflavin T Seeding Experiments

Fibril seeds were prepared as previously described [64]. In brief, 10 mg/mL lyophilized native ac-αSyn was dissolved in PBS in a microcentrifuge tube and shaken at 300 rpm for 5 days without a Teflon bead. The resulting solution was then centrifuged at 10 k rpm for 30 min and resuspended with PBS twice to ensure all monomeric ac-αSyn was removed. Fibril concentration was assessed by dissolving an aliquot of fibrils in 8 M guanidinium hydrochloride and measuring A_280_. In seeded ThT assays, 1 μM of fibril seeds were added to 70 μM of monomeric native or glycated ac-αSyn, 20 μM ThT, in PBS at pH 7.4. Fibril growth was monitored as a function of ThT fluorescence measured every 33 min at 37 °C under quiescent conditions (without shaking). 

### 2.9. UV–vis Spectroscopy

UV–vis wavelength scans of 100 µM native or glycated ac-αSyn in the presence or absence of DJ-1 in PBS, pH 7.4 were acquired in a 1-centimeter quartz cuvette using PBS as the blanking buffer. Initial concentrations were determined using a BCA assay. Absorbance was measured at variable wavelengths from 250–500 nm in increments of 0.5 nm on a UV–vis spectrophotometer. 

### 2.10. Liquid AFM Imaging

AFM images were acquired on a Cypher ES AFM (Asylum Research) using PNP-DB tips with a nominal spring constant of ≈0.5 N/m and drive frequency of ≈67 kHz. For sample preparation, mica was first treated in an aminopropyl silatrane (APS) solution (67 µM) for 30 min to functionalize the surface and then washed thoroughly with ultrapure water and PBS. The samples were then deposited onto the surface (50 µL droplet, all samples had a concentration of 10 µg/mL) and allowed to bind for 20 min at room temperature. The surface was then washed again with PBS and placed into the AFM for imaging. Care was taken to ensure the samples were never allowed to dry. All images were taken at room temperature in standard tapping mode with a resolution of 256 × 256 pixels. Additionally, blueDrive (Asylum Research, Oxford Instruments) photothermal excitation was utilized to ensure high quality imaging in liquid conditions. 

Images obtained were processed using the “Particle Analysis” function in the Asylum Research AFM software, which yielded heights for all particles in the images. These values were then grouped by sample and exported into MATLAB (R2020b). A one-way analysis of variance (ANOVA) was then performed on the grouped data to determine significant differences. 

## 3. Results

### 3.1. Lysine-Rich N-Terminus and NAC of ac-αSyn Are Most Susceptible to Glycation Effects

**Figure 1:**
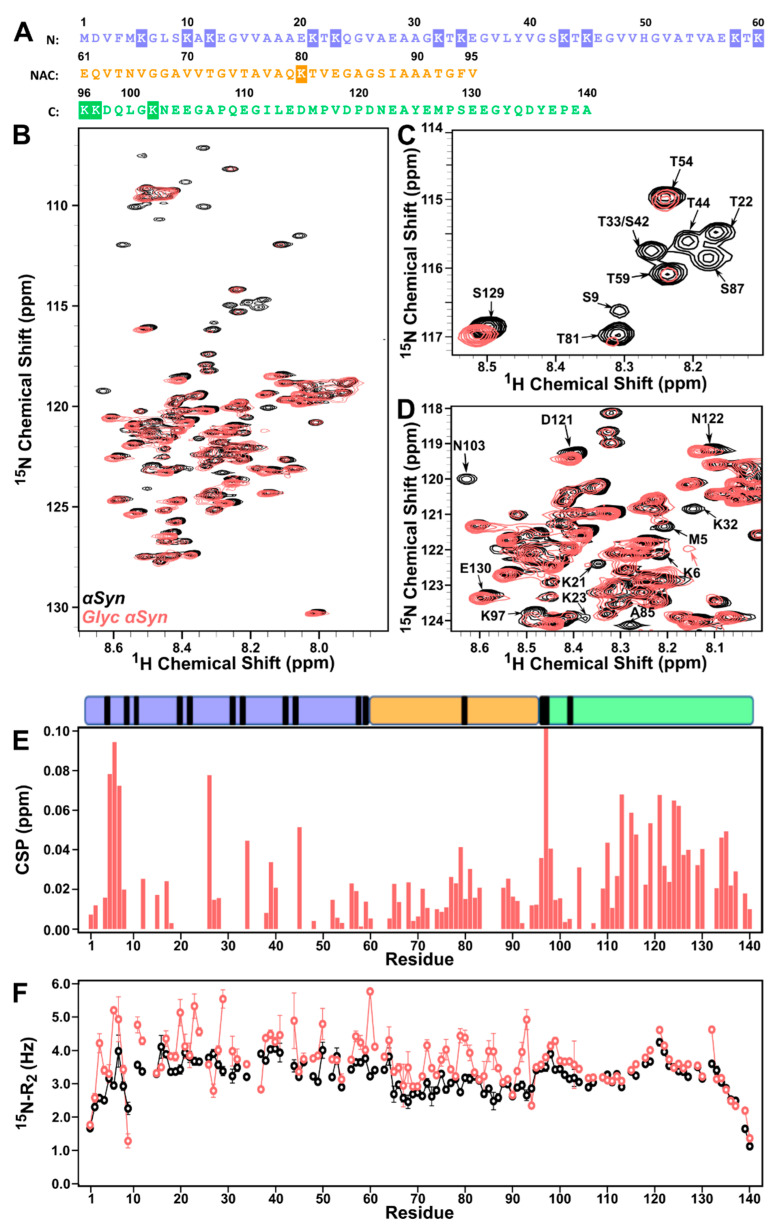
Residue-specific dynamic differences between native ac-αSyn and glyc-ac-αSyn. (**A**) Primary sequence of ac-αSyn, highlighting the lysines as potential glycation sites for MGO (purple—N-terminal, orange—NAC, and green—C-terminal domains). (**B**) ^1^H–^15^N HSQC spectra of native ac-αSyn (black) and glyc-ac-αSyn (red) indicating the intrinsically disordered structure of ac-αSyn even upon glycation. (C–D) Zoomed-in regions of the ^1^H–^15^N HSQC shown in (**B**) of (**C**) the serine and threonine region, which shows a dramatic drop in peak intensities upon glycation, and (**D**) a region that highlights chemical shift perturbations (CSPs) of C-terminal residues upon glycation. (**E**) The chemical shift perturbations of glyc-ac-αSyn relative to native ac-αSyn show that perturbations are localized in the early N-terminus and C-terminus. The three domains of ac-αSyn are shown at the top and color-coded as in (**A**). Lysines are highlighted with black boxes. (**F**) The ^15^N transverse relaxation rates (^15^N-R_2_) of native ac-αSyn (black) and glyc-ac-αSyn (red) show decreased dynamics in the N-terminus and NAC, near lysine residues. C-terminal residues show indistinguishable ^15^N-R_2_ values between native and glyc-ac-αSyn. Error bars are determined from the fitting errors of the single exponential decay fits. All spectra were acquired on 250 µM ac-αSyn in PBS at 15 °C.

Glycated ac-αSyn (glyc-ac-αSyn) was produced from the reaction of ac-αSyn with MGO. The resulting glyc-ac-αSyn is distinct from native ac-αSyn in chemical composition, as assessed using the UV–Vis absorbance profile, and aggregation characteristics, evaluated from size exclusion chromatography (SEC) and thioflavin T (ThT) fluorescence) (Figure S2). The SEC chromatogram shows the presence of small oligomers (~13–16 mL elution) that form due to the glycation of ac-αSyn and are not present in native ac-αSyn. In order to determine the residue-specific effects of glycation on ac-αSyn, we used solution NMR to investigate residue specific perturbations to structure and dynamics of ^15^N-ac-αSyn monomers in solution upon glycation with MGO. Native αSyn consists of 15 lysines that are susceptible to MGO-mediated glycation, several of which are part of the imperfect KTKEGV repeats that are concentrated in the N-terminus (Figure 1A). The ^1^H–^15^N HSQC (heteronuclear single quantum coherence) spectrum of ^15^N-glyc-ac-αSyn (Figure 1B) shows that observed resonances have a significant peak overlap with the native form of ac-αSyn. The ^15^N-glyc-ac-αSyn sample contains a mixture of monomers and non-isolatable oligomers observed in SEC. However, the oligomers likely tumble too slowly in solution to be detected by solution NMR. The considerable resonance overlap with native ac-αSyn indicates that we are observing monomeric glyc-ac-αSyn in the ^1^H–^15^N HSQC and that it maintains an intrinsically disordered structure and a similar conformational ensemble to native ac-αSyn. However, substantial peak intensity losses with increased ^15^N-transverse relaxation rates (R_2_) are observed in the lysine-rich N-terminal and the non-amyloidβ component (NAC) regions of glyc-ac-αSyn (Figure 1C) and small chemical shift perturbations are observed in C-terminal residues (Figure 1D,E). The significant broadening observed in N-terminal and NAC residues suggests that this region is undergoing intermediate exchange, which may arise due to the chemical glycation, conformational changes, and/or interactions between the glyc-ac-αSyn monomers and undetectable oligomers in solution that are induced by the glycation reaction. A slight increase in ^15^N-R_2_ values is observed in the C-terminus of ^15^N-glyc-ac-αSyn only up to residue 106, four residues past the most C-terminal lysine, K102, with less substantial peak intensity losses near the C-terminal lysines compared to the N-terminal and NAC regions. However, the observation of chemical shift perturbations in the C-terminus indicates that this region experiences fast exchange, which may occur due to conformational changes in the protein or weak interactions. Thus, the N-terminal and NAC regions are most susceptible to glycation effects caused by the MGO reaction.

### 3.2. DJ-1 Restores Native-like Character to Glyc-ac-αSyn

**Figure 2:**
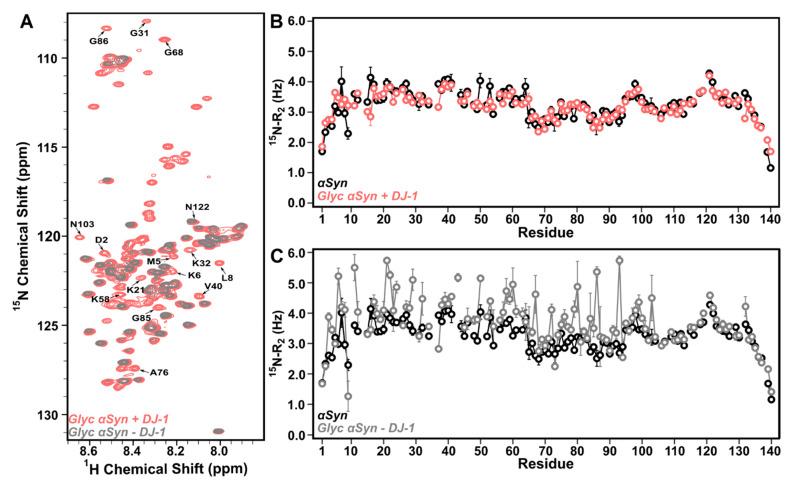
DJ-1 restores native-like character to glyc-ac-αSyn. (**A**) ^1^H–^15^N HSQC spectra of 250 µM ^15^N-glyc-ac-αSyn in the presence of 250 µM DJ-1 (red) or upon removal of DJ-1 (grey) show that peak intensities are recovered when DJ-1 is present in the sample (red) but are reduced to the levels of glyc-ac-αSyn alone once DJ-1 is removed from the sample (grey). (**B**) ^15^N-R_2_ values of 250 µM ^15^N-native ac-αSyn alone (black) or 250 µM ^15^N-glyc-ac-αSyn in the presence of DJ-1 (red). The backbone dynamics of glyc-ac-αSyn with DJ-1 (red) largely overlap with native ac-αSyn without DJ-1 (black), consistent with the peak intensities in the ^1^H–^15^N HSQC. (**C**) Upon removal of DJ-1 (grey), the backbone dynamics as measured by ^15^N-R_2_ rates revert back to resembling those of glyc-ac-αSyn alone, displaying increased ^15^N-R_2_ in the N-terminal and NAC regions relative to native ac-αSyn (black). Error bars are determined from the fitting errors of the single exponential decay fits. All samples were monitored in PBS at 15 °C.

DJ-1 has been shown to mitigate the effects of MGO on free amino acids as a glyoxalase [33]; however, mechanistic details of how DJ-1 impacts glycation of larger proteins remains controversial [44,65]. Although, there is some evidence that it may deglycate small compounds and free amino acids [42,66]. In order to determine the residue-specific effect of DJ-1 on glyc-ac-αSyn, we monitored ^15^N-glyc-ac-αSyn using NMR upon addition of DJ-1. Upon incubation of ^15^N-glyc-ac-αSyn with a 1:1 molar ratio of DJ-1, surprisingly, residue specific chemical shifts and peak intensities of ^15^N-glyc-ac-αSyn in ^1^H–^15^N HSQC spectra are now similar to those of native ac-αSyn (Figure 2A, red and Figure S3), and ^15^N-R_2_ values are also indistinguishable from ^15^N-native ac-αSyn (Figure 2B). This suggests that the interaction of DJ-1 with glyc-ac-αSyn restores structural and dynamic characteristics of native ac-αSyn to glyc-ac-αSyn either through chemical deglycation, or by suppressing conformational exchange of the glyc-ac-αSyn protein.

To further investigate the role of DJ-1 on glyc-ac-αSyn, we removed DJ-1 from the solution to determine whether the modification was permanent or due to DJ-1–glyc-ac-αSyn interactions. Strikingly, upon filtration of DJ-1 from solution, the ^15^N-glyc-ac-αSyn ^1^H–^15^N HSQC spectrum reverts back to its original signature and shows peak intensities, linewidths, and chemical shifts that are indistinguishable from glyc-ac-αSyn before the addition of DJ-1 (Figure 2C). This indicates that DJ-1 does not chemically deglycate glyc-ac-αSyn, since the deglycated ac-αSyn could not be spontaneously glycated without the presence of MGO or another glycating agent. These data are supported by UV–Vis absorbance spectra, which show no substantial change in the absorbance wavelength profile between glyc-ac-αSyn in the absence of DJ-1 and after removal of DJ-1, presenting the characteristic increased absorbance from ~300–400 nm relative to native ac-αSyn (Figure S4). In addition, the reaction of DJ-1 with glyc-ac-αSyn does not enable amyloid formation, as assessed using ThT fluorescence, as would be expected if the ac-αSyn was chemically deglycated by DJ-1 (Figure S5). Together, these data suggest that DJ-1 does not effectively deglycate ac-αSyn, but rather imposes native-like structural and dynamic characteristics on glyc-ac-αSyn in solution via protein–protein interactions. 

### 3.3. DJ-1 Interacts Primarily with Glyc-ac-αSyn Oligomers via Its Catalytic Site

**Figure 3:**
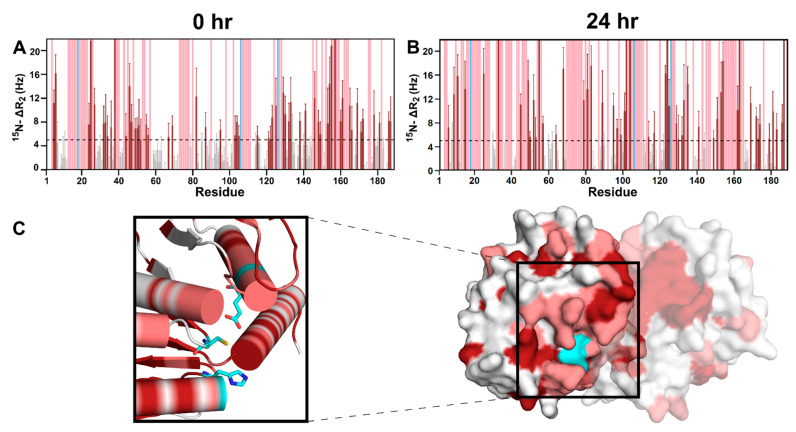
The catalytic site of DJ-1 is an interaction interface for glyc-ac-αSyn. (**A**) Per-residue ΔR_2_ values of ^15^N DJ-1 in the presence of glyc-ac-αSyn with 0-hour pre-incubation (no pre-incubation) showing residue-specific R_2_ enhancement (dark red) or peak broadening beyond detection (light red); cyan residues indicate the catalytic triad. (**B**) ^15^N-ΔR_2_ values of ^15^N-DJ-1 with glyc-ac-αSyn that had been pre-incubated at room temperature for 24 h to increase the concentration of oligomers in solution. The increased ^15^N-ΔR_2_ of DJ-1 in the presence of a higher concentration of glyc-ac-αSyn oligomers indicates that DJ-1 has greater binding propensity to ac-αSyn oligomers than monomers. All residues in grey are under 5 Hz change. (**C**) ^15^N-ΔR_2_ values of DJ-1 in the presence of glyc-ac-αSyn with 0-hour pre-incubation are mapped onto the 3D structure of DJ-1 (colored as in **A**), highlighting the interaction site near the catalytic triad of DJ-1. The catalytic triad is shown in cyan. All spectra were collected using a 700 MHz spectrometer at 25 °C.

In order to further characterize the specificity of the DJ-1–glyc-ac-αSyn interactions and determine a binding interface on DJ-1, we monitored ^15^N-DJ-1 chemical shift, peak intensity, and ^15^N-R_2_ changes upon incubation with glyc-ac-αSyn using NMR spectroscopy. Upon incubating ^15^N-DJ-1 with glyc-ac-αSyn, residue-specific ^15^N-R_2_ increases and significant line broadening are observed (Figure 3A,B). No chemical shift perturbations are observed. By mapping the residues with significant increases in ^15^N-R_2_ (Figure 3A,B, dark red) and/or line broadening (Figure 3A,B, light red) on the dimer structure of DJ-1, it is apparent that perturbations due to the presence of glyc-ac-αSyn are located primarily in the catalytic triad and surrounding residues (Figure 3C). The same interaction site on DJ-1 was also observed for interaction with native ac-αSyn (Figure S6A,B).

To assess the necessity of the catalytic triad for the DJ-1–ac-αSyn interaction, the catalytic site C106 was either mutated to alanine or not oxidized. Upon addition of ac-αSyn to C106A-DJ-1 or non-oxidized DJ-1, the ^15^N-R_2_ increases seen in WT-DJ-1 are completely abolished (Figure S6C,D), suggesting that interaction of DJ-1 with ac-αSyn does not arise under these conditions. In addition, the C106A mutation or the cysteine reduction in DJ-1 dramatically suppresses ac-αSyn amyloid inhibition by DJ-1 (Figure S7). Together, these data support the role of the DJ-1 catalytic active site in the DJ-1–ac-αSyn interaction.

We probed the DJ-1 interactions with native ac-αSyn from the perspective of ac-αSyn monomers by monitoring ^15^N-native ac-αSyn monomer NMR signals upon the addition of DJ-1. However, the co-incubation of ^15^N-native ac-αSyn with DJ-1 in a 1:2 molar ratio resulted in no significant changes in line broadening or chemical shifts (Figure S8), despite changes in the ^15^N-R_2_ values of DJ-1 described above. One explanation for this is that the DJ-1 is interacting primarily with ac-αSyn oligomers rather than monomers. Thus, while we observe perturbations to DJ-1 due its interactions with native or glycated ac-αSyn oligomers, there is no observable effect on the unperturbed native ac-αSyn monomers. 

**Figure 4:**
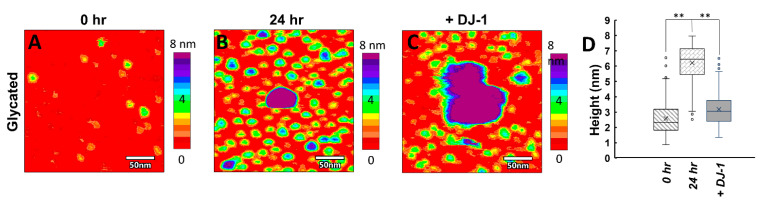
Liquid AFM of glyc-ac-αSyn highlighting changes in observed species upon incubation with DJ-1. (**A**) Freshly prepared glyc-ac-αSyn primarily presents as circular, compact monomers ≈3 nm in height (yellow–green). (**B**) After 24 h of incubation at room temperature, smaller oligomeric species ≈6 nm in height (green–blue) in addition to larger oligomers over 8 nm in height (purple) are apparent. (**C**) When DJ-1 is introduced to glyc-ac-αSyn that had been pre-incubated for 24 h, the aggregates coalesce, forming large, amorphous segmented clusters. However, monomers are left free on the surface, indicating that the oligomeric species are sequestered into the DJ-1 induced complexes, while monomers are left free in solution. (**D**) Distributions of heights from the above AFM samples. For this calculation only objects in the images below 8 nm in height were accounted for (0 h = 2.58 ± 1.04 nm, N = 432; 24 h = 6.21 ± 1.17 nm, N = 596; +DJ1 = 3.18 ± 1.00, N = 423). ** denotes *p*-values << 0.01.

To support this argument, we increased the concentration of glycated or native ac-αSyn oligomers added to ^15^N-DJ-1 and monitored changes in DJ-1 NMR signals. Incubating glyc-ac-αSyn at room temperature for 24 h resulted in increased amounts of oligomers in the sample as observed using AFM imaging (Figure 4A,B,D). Indeed, with increased concentrations of glycated or native ac-αSyn oligomers, DJ-1 shows increased ^15^N-R_2_ values and peak broadening to more residues (Figure 3B and Figure S6B, compared to Figure 3A, Figure S6A), suggesting that DJ-1 primarily interacts with ac-αSyn oligomers. 

### 3.4. Glyc-ac-αSyn Oligomers Participate in DJ-1 Induced Complexes, Releasing Monomers in Solution

In order to directly observe how the glyc-ac-αSyn oligomer and monomer species are perturbed upon addition of DJ-1, we used AFM in solution. Alone in solution, glyc-ac-αSyn that has been preincubated at room temperature for 24 h exists as monomeric species ~3 nm (Figure 4, green), smaller oligomers ~6–8 nm (Figure 4, blue), and larger oligomers > 8 nm (Figure 4, purple). Co-incubation of DJ-1 with preincubated glyc-ac-αSyn results in amorphous, segmented complexes (Figure 4C) that are larger than either glyc-ac-αSyn oligomers or DJ-1 in height and area. Strikingly, the addition of DJ-1 significantly reduces the number of glyc-ac-αSyn oligomers on the order of ~6–8 nm that are observed in the AFM images (Figure 4B–D), supporting their uptake into the DJ-1-induced complexes that are larger than 8 nm. Meanwhile, monomers ~3 nm in height (Figure 4, green) remain free and dispersed on the substrate, suggesting that they do not participate in the complexes. A quantitative boxplot distribution analysis of particle heights under these conditions (Figure 4D) directly demonstrates that DJ-1 interacts with oligomers while leaving the monomer glyc-ac-αSyn free in the solution. The AFM data are consistent with our NMR data in Figure 2B, which shows that upon addition of DJ-1 to glyc-ac-αSyn, the ^15^N-R_2_ rates of glyc-ac-αSyn revert to native-like values, representative of free ac-αSyn monomers in solution. These data support our hypothesis that glycated and native ac-αSyn oligomers interact with DJ-1, while monomers do not participate in the interactions and remain free in solution. Notably, the interaction of DJ-1 with glyc-ac-αSyn oligomers does not degrade the oligomers, as evidenced by the restoration of enhanced ^15^N-R_2_ rates upon removal of DJ-1 that are indistinguishable from those in the absence of DJ-1 (Figure 2C). 

## 4. Discussion

The glycation of proteins has been shown to lead to increased protein aggregation and hindered cellular clearance and is associated with degenerative diseases such as PD, Alzheimer’s disease, diabetes, and atherosclerosis. The glycation of αSyn has been shown to increase oligomer formation and produce heterogeneous amorphous aggregates and suppress amyloid formation [10,31]. Investigating the changes induced by glycation on the biophysical characteristics of αSyn can help clarify the role of glyc-αSyn in synucleinopathies. Our SEC data and AFM imaging are consistent with the literature, showing a new population of oligomers formed by glyc-ac-αSyn. Although glyc-ac-αSyn does not form amyloid fibrils, the aggregates that it does produce are toxic to cells, are unable to be cleared, and alter lipid binding to disrupt physiological function [32,67,68,69]. Understanding how to interfere with these toxic oligomers may aid in therapeutic design against pathological glycation. 

DJ-1 is known to interact with and regulate numerous proteins implicated across various biological systems, including neurodegenerative disorders [46,70,71], diabetes [72], and cancer [73]. Numerous studies have addressed interactions of DJ-1 with various forms of native αSyn, including with monomer, oligomer, and amyloid species [49,52,55,74]. DJ-1 has been reported to have weak to minimal binding to αSyn monomers [55] and while direct interactions with αSyn oligomers have not been established in vitro [52], DJ-1 has been shown to reduce αSyn oligomerization in vivo [49,74]. In addition, DJ-1 has been shown to attenuate αSyn aggregation through chaperone-mediated autophagy [54]. Prior research shows that DJ-1 binds to aggregated forms of αSyn and that αSyn fibrils have increased toxicity following alteration by DJ-1, indicating that DJ-1 modifies aggregated forms of αSyn [53]. 

Here, we are interested in understanding the molecular interactions of DJ-1 with glyc-ac-αSyn monomers and oligomers. Very little is known about DJ-1 interactions with glycated proteins. However, DJ-1 has been proposed to act as a glyoxalase to help reduce the harmful effects of glycating species [33] and overexpression of DJ-1 has been shown to mitigate the glycation-induced toxicity and aggregation of αSyn [57]. Glycated αSyn monomers and oligomers can induce an increase in oxidative stress leading to further toxicity [23]. The upregulation of DJ-1 has been proven to mitigate the effects of oxidative stress by acting as a scavenger for reactive oxygen species [75,76,77]. Therefore, controlling DJ-1 expression is essential to reducing αSyn toxicity caused by the effects of ROS and glycation.

**Figure 5:**
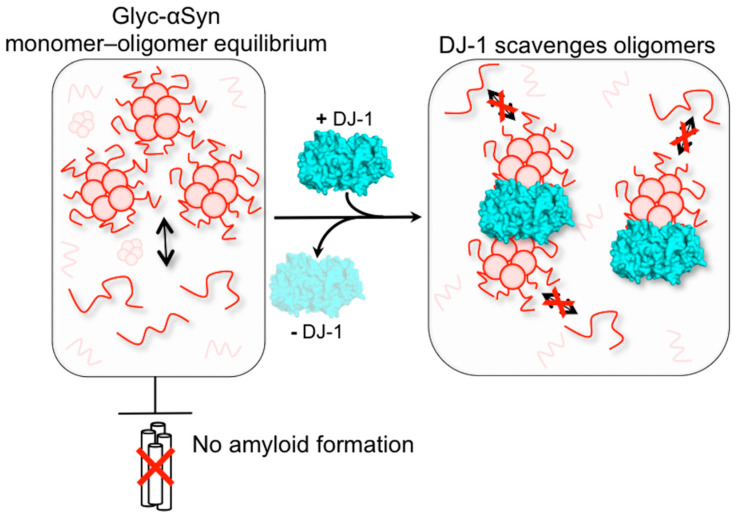
Proposed mechanism for DJ-1’s impact on ac-αSyn glycation. (Left) Glyc-ac-αSyn spontaneously forms oligomers that are in equilibrium with glyc-ac-αSyn monomers. Glyc-ac-αSyn does not form amyloid fibrils, although the oligomers have been shown to be harmful to neurons. (Right) Upon addition of DJ-1, DJ-1 scavenges glyc-ac-αSyn oligomers, preventing their interactions with glyc-ac-αSyn monomers. This allows glyc-ac-αSyn monomers to be free in solution. Removal of DJ-1 from the system restores the glyc-ac-αSyn monomer–oligomer interactions.

Based on the combination of AFM and NMR results, we propose that (1) DJ-1 interacts with glyc-ac-αSyn oligomers to sequester them into larger aggregates, (2) that the sequestration of oligomers by DJ-1 suppresses glyc-ac-αSyn monomer–oligomer interactions, and (3) that the sequestration of oligomers allows the release of free glyc-ac-αSyn monomers (Figure 5). By inhibiting these monomer–oligomer interactions, the aggregation and propagation of αSyn aggregates may be suppressed, providing an avenue for therapeutic intervention to mitigate the harmful effects of the aging-induced glycation of αSyn. We demonstrate that the catalytic triad of DJ-1, specifically C106, is largely responsible for the interaction with ac-αSyn oligomers supporting previous research on the oxidation state of DJ-1 that shows that the proper oxidation of C106 to the sulfinic acid form is imperative for function as a chaperone, its redox capability, and its cytoprotective function [38,78,79,80]. 

DJ-1 expression and oxidation levels can potentially be biomarkers for progressive forms of Parkinson’s disease [81,82,83]. The upregulation of DJ-1 has been proven to mitigate the effects of oxidative stress by acting as a scavenger for reactive oxygen species [75,76,77]. Therefore, large amounts of ROS may alter the oxidation state of DJ-1 within the cell and lead to decreased DJ-1 function as a modulator of αSyn aggregation. As a consequence of these DJ-1–glyc-ac-αSyn interactions found in our study, a decreased accumulation of glyc-ac-αSyn aggregates within dopaminergic neurons may protect against neurodegenerative effects caused by harmful αSyn aggregates. Thus, targeting the upregulation of DJ-1 and suppressing the effects caused by αSyn glycation and amyloid accumulation may aid in treatment strategies against Parkinson’s disease.

## Data Availability

Data are available upon reasonable request from the corresponding author.

## Supplementary Materials

The following are available online at www.mdpi.com/article/10.3390/biom11101466/s1, Figure S1: Impact of DJ-1 on native αSyn amyloid formation, Figure S2: Characterization of glyc-αSyn, Figure S3: DJ-1 restores native-like features in ^1^H–^15^N HSQC spectra, Figure S4: Incubation with DJ-1 does not deglycate αSyn, Figure S5: Impact of αSyn glycation on amyloid formation, Figure S6: Supporting the DJ-1 catalytic site as the binding site for αSyn, Figure S7: Impact of the DJ-1 catalytic triad on αSyn amyloid formation, and Figure S8: Impact of DJ-1 on αSyn monomers. References [10,32,51,52,53] are also cited in the Supplementary Materials.
