# Supplementary material for: DJ-1 Acts as a Scavenger of α-Synuclein Oligomers and Restores Monomeric Glycated α-Synuclein
Source: Biomolecules. 2021 Oct 6;11(10):1466. doi: 10.3390/biom11101466 (PMC8533443; doi:10.3390/biom11101466)
Supplement: Supplementary file 1 [file biomolecules-11-01466-s001.zip › biomolecules-1376162-supplementary.pdf]

Supplementary Materials for

DJ-1 acts as a scavenger of α-synuclein oligomers and restores monomeric glycated α-synuclein

Tamr B. Atieh, Jonathan Roth, Xue Yang, Cody L. Hoop, and Jean Baum *

Department of Chemistry and Chemical Biology, Rutgers University, Piscataway, New Jersey 08854

* Corresponding author: Jean Baum; Dept. of Chemistry and Chemical Biology, Rutgers University, 123 Bevier Rd., Piscataway, NJ 08854; Tel.: 848-445-5284; Email: jean.baum@chem.rutgers.edu

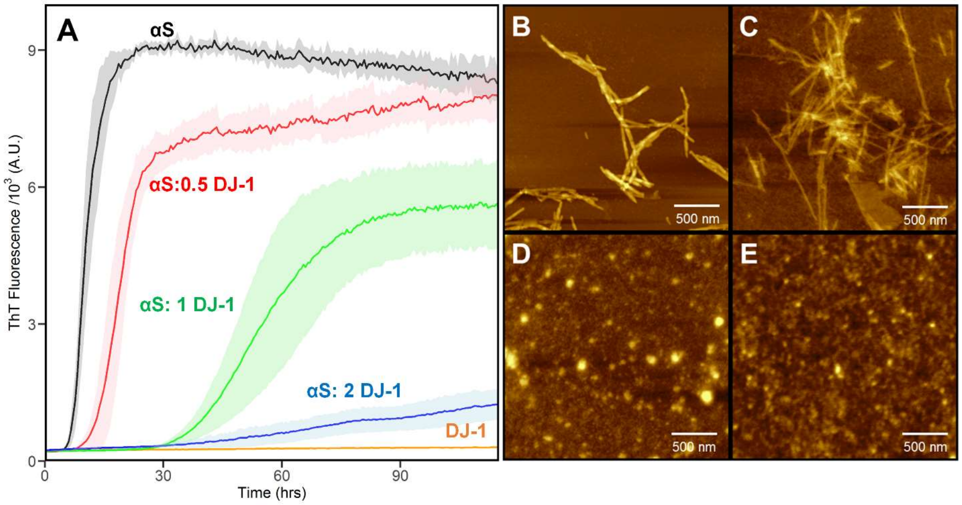

Figure S1. Impact of DJ-1 on native ac-αSyn amyloid formation. (A) ThT fluorescence of 70 μM native ac-αSyn alone (black) or in the presence of increasing concentrations of oxidized DJ-1 (dimer concentrations- 35 μM, red; 70 μM, green; or 140 μM, blue). ThT fluorescence of DJ-1 alone is shown in orange. Addition of DJ-1 to native ac-αSyn extends the lag time from 5–10 hours in the absence of DJ-1 to 20–50 hours with 35–70 μM DJ-1. At 140 μM DJ-1, ac-αSyn fibrillization is completely inhibited in this time regime. These results are consistent with and supports previous work, which shows that DJ-1 suppresses amyloid formation of αSyn [51,52,53]. (B–E) AFM images of (B) ac-αSyn alone, (C–D) ac-αSyn incubated with DJ-1 in a (C) 1:1 or (D) 1:2 molar ratio of ac-αSyn monomer to DJ-1 dimer, respectively, or (E) DJ-1 alone, taken from the end of the ThT fluorescence assay. ac-αSyn fibrils are observed in the absence of DJ-1 (B) and with substoichiometric concentrations of DJ-1 (C). However, ac-αSyn fibrils are less prevalent with higher concentrations of DJ-1 (D), supporting amyloid inhibition. AFM images were acquired in air using a Park NX-10 AFM. 20 μL of sample were deposited on a 1 cm x 1 cm freshly cleaved mica and incubated for 10 minutes. Samples were then washed with 200 μL water to remove excess salt and unbound protein and allowed to dry for 1 hour before imaging. AFM images were processed using Gwyddion software.

Assessing methylglyoxyl-mediated glycation of N-terminally acetylated α-synuclein.

To determine if glycated N-terminally acetylated α-synuclein (glyc-ac-αSyn) was produced from reaction with methylglyoxyl (MGO), we assayed the chemical composition and aggregation propensity of the MGO-glycated ac-αSyn. By reacting ac-αSyn with MGO, we detect glycation as evidenced by a visual change in color from a colorless to brown solution along with an increase in absorbance from ~300–500 nm relative to untreated ac-αSyn in a UV-Vis absorbance wavelength scan (**Figure S2A**). Further attempts to isolate the monomer via size exclusion chromatography (SEC) reveal that glyc-ac-αSyn contains an intrinsic population of oligomeric species not present in untreated ac-αSyn (**Figure S2B**). These oligomers, however, do not propagate into amyloid fibrils, as evaluated by the lack of Thioflavin T (ThT) fluorescence of glyc-ac-αSyn within 80 hours (**Figure S2C**). This is consistent with previous studies, which show that glycation of αSyn abrogates amyloid formation [10,32]. By contrast, untreated ac-αSyn forms filamentous fibrils with heights ranging from 5–15 nm with various lengths up to 2 μm (**Figure S1B**).

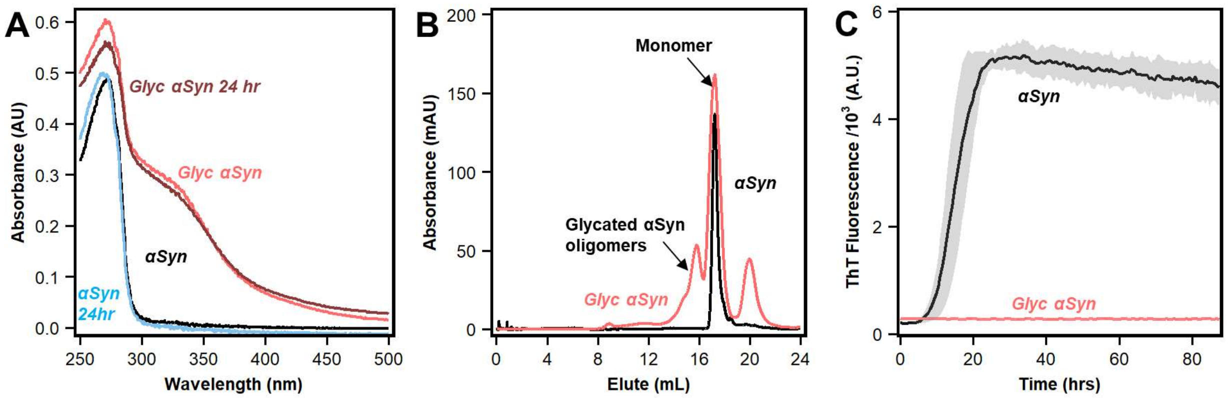

Figure S2. **Characterization of glyc-ac-αSyn.** (A) UV-Vis spectra of fresh native ac-αSyn (black), fresh glyc-ac-αSyn (red), and native (blue) and glyc-ac-αSyn (dark red) after incubation at room temperature for 24 hrs. The glycation of ac-αSyn results in a striking increase in absorbance between 300–500 nm. Incubation of ac-αSyn, which increases production of oligomers, does not perturb the UV-Vis profile of native ac-αSyn or glyc-ac-αSyn. (B) Size exclusion chromatograms of native (black) and glyc-ac-αSyn (red). Both samples show a sharp monomer peak near 17 mL elution time. However, glyc-ac-αSyn displays a peak between 13–16 mL, indicating existence of intrinsic oligomers. (C) ThT fluorescence curves of native ac-αSyn (black) show characteristic fibril formation, while glycation of ac-αSyn (red) abolishes amyloid aggregation.

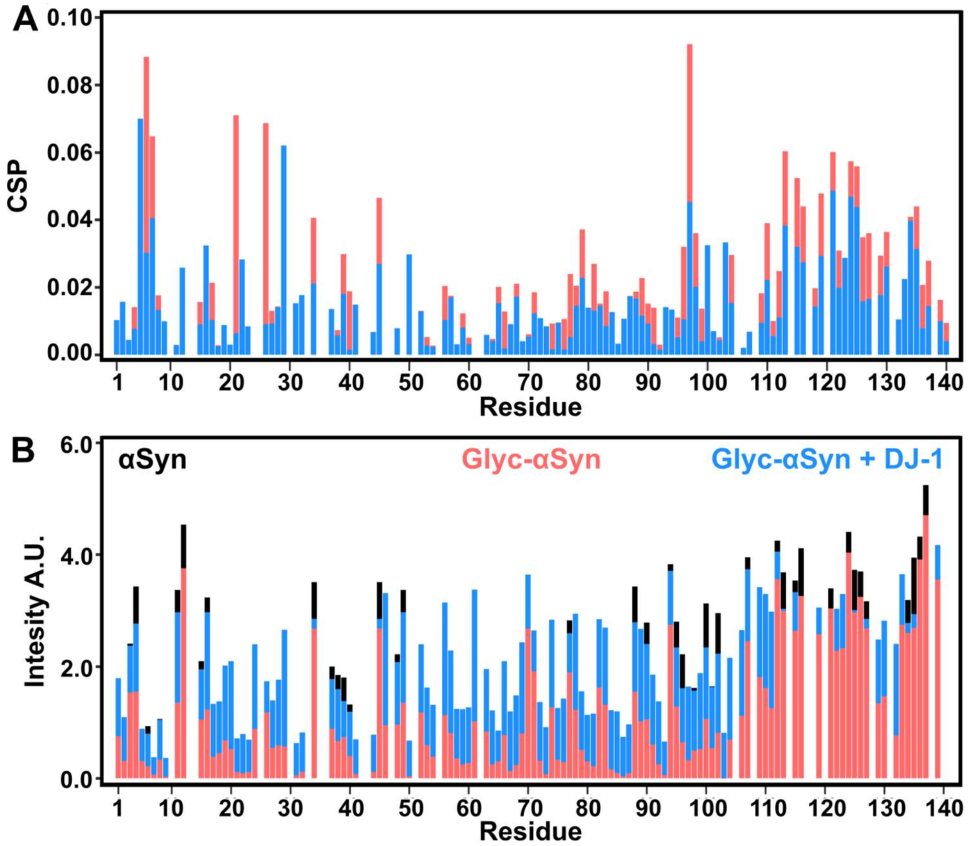

Figure S3. DJ-1 restores native-like features in ^{1}H–^{15}N HSQC spectra. (A) Combined chemical shift perturbations of glyc-ac-αSyn in the presence (blue) or absence (red- reproduced from Fig. 1E) of DJ-1 relative to native ac-αSyn. Chemical shift changes incurred by glycation are reduced, but not fully recovered upon addition of DJ-1, consistent with ac-αSyn remaining glycated even in the presence of DJ-1. (B) Per-residue peak intensities of native ac-αSyn (black), glyc-ac-αSyn in the absence of DJ-1 (red), or glyc-ac-αSyn in the presence of DJ-1 (blue). Peak intensities largely recover with DJ-1 present in the sample.

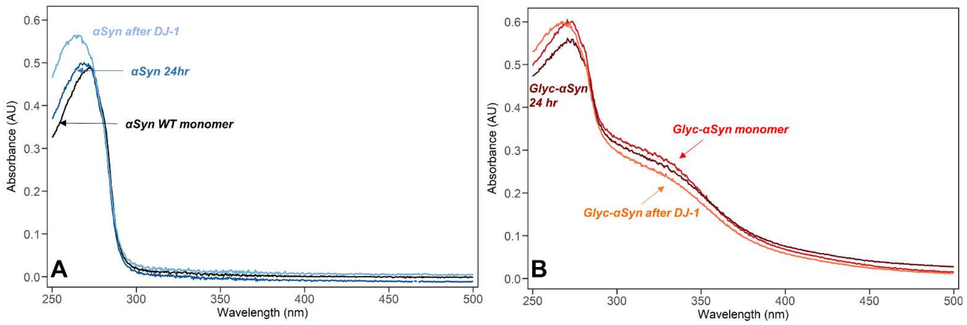

Figure S4. Incubation with DJ-1 does not deglycate ac-αSyn. (A) UV-Vis wavelength profiles of fresh native ac-αSyn monomer (black), after incubation at room temperature for 24 hr (blue), or upon incubation with DJ-1 (light blue). All show a consistent wavelength profile with the maximum absorbance near 280 nm. (B) Freshly glycated ac-αSyn monomer (red) shows increase in absorbance from 300 to 500 nm, relative to native ac-αSyn, indicative of modification induced by glycation. The absorbance at these wavelengths remains unchanged after incubation at room temperature for 24 hr (dark red) or upon incubation with DJ-1 (orange).

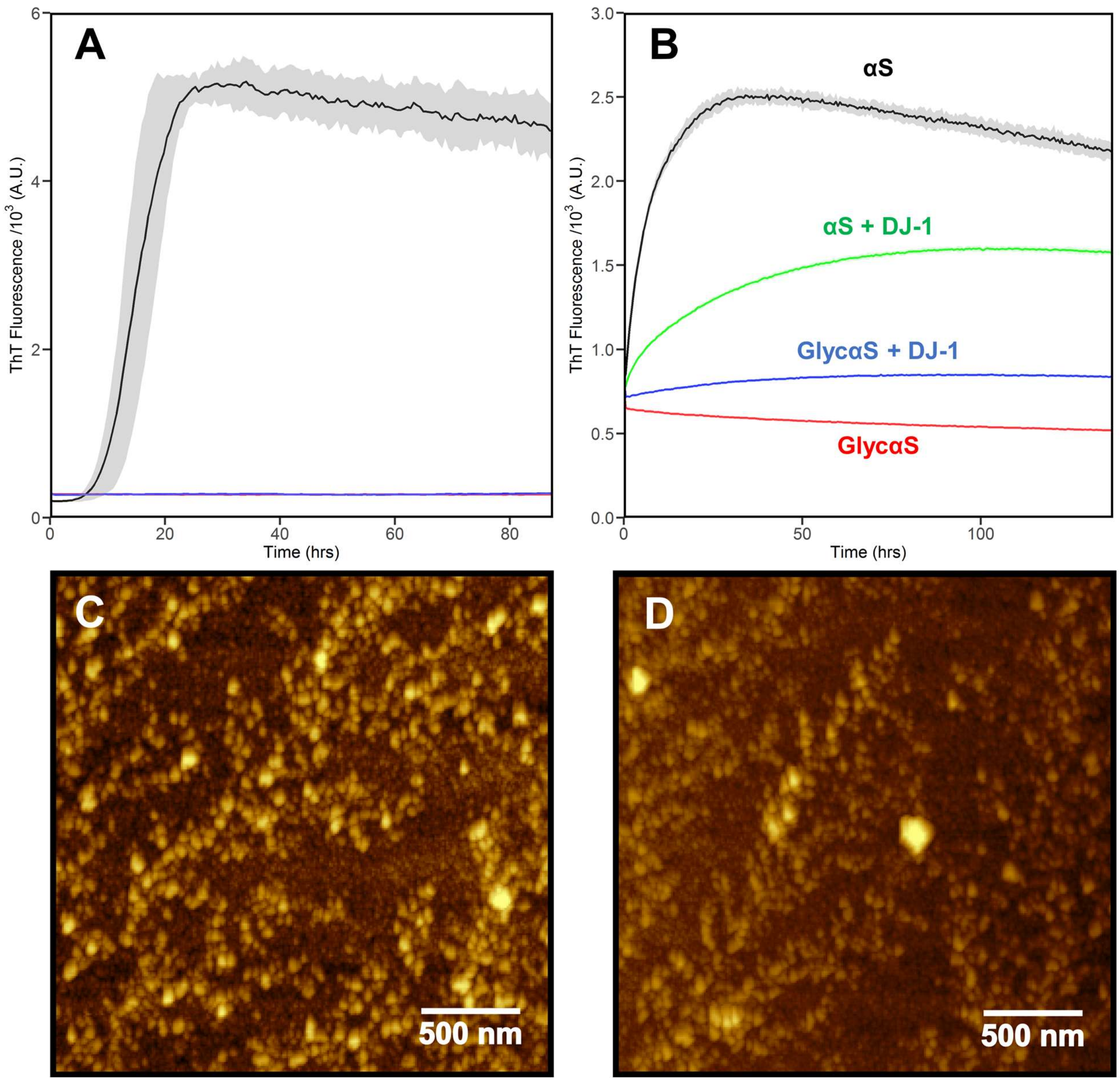

Figure S5. Impact of ac-αSyn glycation on amyloid formation. (A) ThT fluorescence of 70 μM native ac-αSyn (black) shows a sigmoidal increase in fluorescence intensity, characteristic of amyloid fibril formation. Glyc-ac-αSyn (70 μM; red) does not show any increase in ThT fluorescence intensity, indicating no amyloid formation. Glyc-ac-αSyn (70 μM) incubated with 35 μM DJ-1 (blue) shows that DJ-1 does not rescue the fibrillization propensity of glyc-ac-αSyn, supporting that deglycation does not occur. DJ-1 was removed from the reaction before performing ThT fluorescence measurements. Assays were performed with 70 μM native or glyc-ac-αSyn monomer with agitation. (B) ThT fluorescence assays under fibril seeding, quiescent conditions. (Black) Native ac-αSyn monomer (70 μM) with 5 μM fibril seeds; (green) native ac-αSyn monomer (70 μM) with 5 μM fibril seeds in the presence of 20 μM DJ-1; (red) glyc-ac-αSyn monomer (70 μM) with 5 μM native ac-αSyn fibril seeds; and (blue) glyc-ac-αSyn monomer (70 μM) with 5 μM native ac-αSyn fibril seeds in the presence of 20 μM DJ-1. DJ-1 suppresses the amyloid growth of native ac-αSyn (green). ThT fluorescence is not observed for glyc-ac-αSyn even in the presence of native ac-αSyn fibril seeds (red), indicating that glyc-ac-αSyn does not template onto native fibril seeds. The addition of DJ-1 to glyc-ac-αSyn (blue) is unable to rescue this cross seeding. All ThT fluorescence assays were performed in PBS, pH 7.4 at 37°C. (C–D) In air AFM image of (C) glyc-ac-αSyn and (D) glyc-ac-αSyn in the presence of DJ-1 at the end point of the ThT assay upon seeding with native ac-αSyn fibrils. No amyloid fibrils are observed for glyc-ac-αSyn in the presence or absence of DJ-1. In air AFM conditions were prepared the same as in Figure S1.

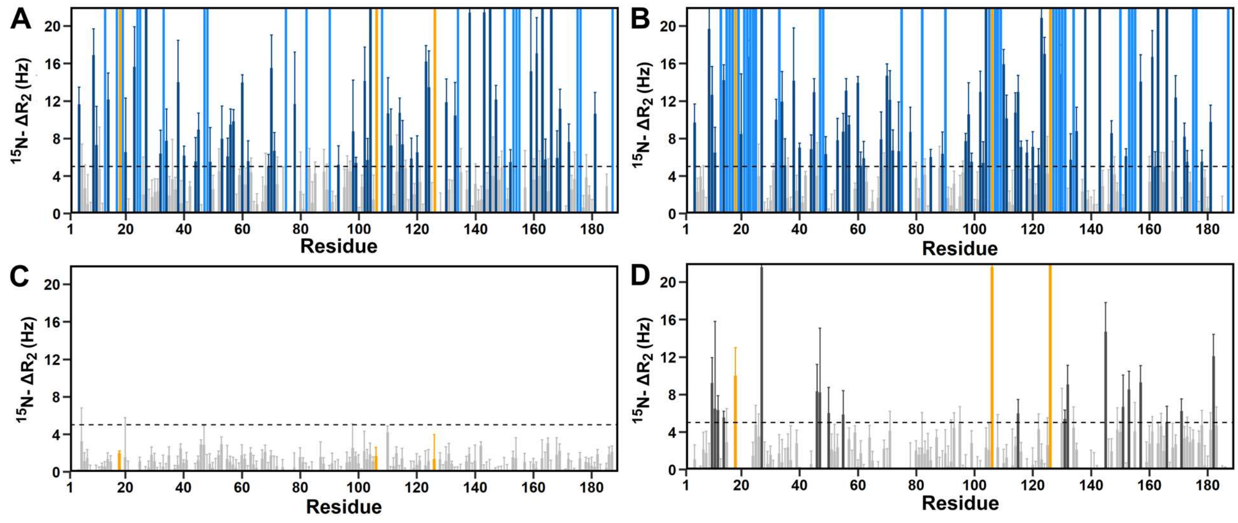

Figure S6. Supporting the DJ-1 catalytic site as the binding site for ac-αSyn. (A–B) Per-residue ^{15}N-ΔR_2 of ^{15}N DJ-1 in the presence relative to in the absence of native ac-αSyn with (A) 0 hours or (B) 24 hours of pre-incubation at room temperature, showing enhanced R_2 values (dark blue) or peak broadening beyond detection (light blue) at specific residues. Orange indicates the catalytic triad, of which all residues are broadened beyond detection. (C) Per-residue ^{15}N-ΔR_2 of ^{15}N-DJ-1/C106A in the presence relative to in the absence of native ac-αSyn. Minimal changes in R_2 in DJ-1/C106A indicate that Cys106 in the catalytic triad is critical for DJ-1–ac-αSyn interactions. (D) Per-residue ^{15}N-ΔR_2 of ^{15}N-DJ-1 in the presence relative to in the absence of native ac-αSyn in non-oxidizing conditions. Substantial increases in R_2 in specific residues in non-oxidizing conditions may result from low, but detectable amounts of oxidized DJ-1 (5% by ESI mass spec, data not shown) present after purification. All ^{15}N-R_2 measurements were performed at 700 MHz ^{1}H Larmor frequency at 25°C in PBS, pH 7.4.

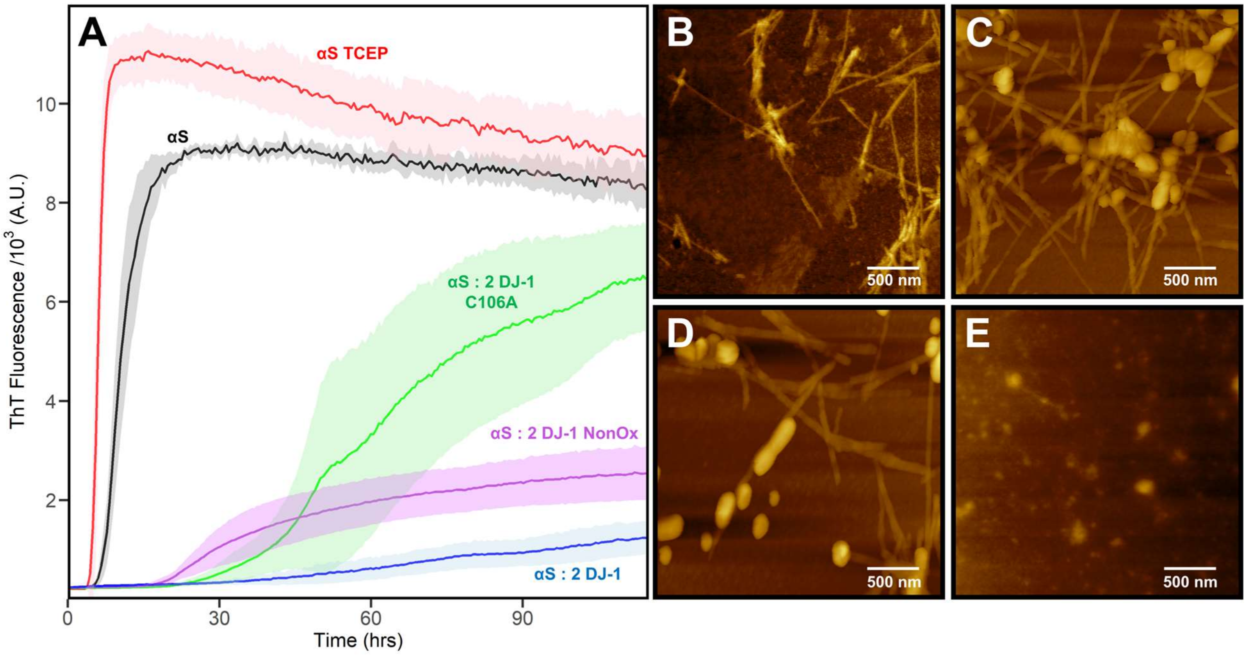

Figure S7. Impact of the DJ-1 catalytic triad on ac-αSyn amyloid formation. (A) ThT fluorescence of (black) native ac-αSyn (70 μM) alone, (red) in the presence of a reducing agent, TCEP, (blue) with addition of 2 molar equivalents of oxidized DJ-1 dimer (140 μM dimer; reproduced from **Fig. S1A**), (magenta) with 2 molar equivalents of DJ-1 dimer and TCEP, and (green) with 2 molar equivalents of DJ-1/C106A dimer. All reactions were assayed in 20 mM sodium phosphate buffer, 100 mM NaCl, at pH 7.4, with 20 μM ThT. All samples (100 μl) were loaded in a 96-well plate with a single Teflon bead in each well, and the plates were shaken at 600 rpm at 37°C for over 100 hours using a POLAR Star Omega fluorimeter to monitor the fluorescence intensity over time. Oxidized DJ-1 is shown to inhibit ac-αSyn amyloid formation at this stoichiometry. By co-incubating ac-αSyn with non-oxidized DJ-1 (addition of a reducing agent or DJ-1/C106A that eliminates the catalytic cysteine), ac-αSyn amyloid formation is delayed by lengthening of the lag time. However inhibition is not as effective as in the presence of oxidized, intact DJ-1. Thus, the presence of C106 and its oxidation state are critical for the DJ-1–ac-αSyn interaction and suppression of ac-αSyn amyloid formation. (B) In air AFM image of ac-αSyn with TCEP that shows similar morphology to native ac-αSyn fibrils. (C) AFM image of ac-αSyn with DJ-1/C106A showing the presence of amyloid fibrils. (D) In air AFM image of ac-αSyn and non-oxidized DJ-1 confirming amyloid fibril formation is not inhibited by the non-oxidized DJ-1. (E) In air AFM image of ac-αSyn and DJ-1 oxidized confirming the complete inhibition of amyloid fibril formation when DJ-1 is properly oxidized. AFM samples were prepared in the same way as **Figure S1**.

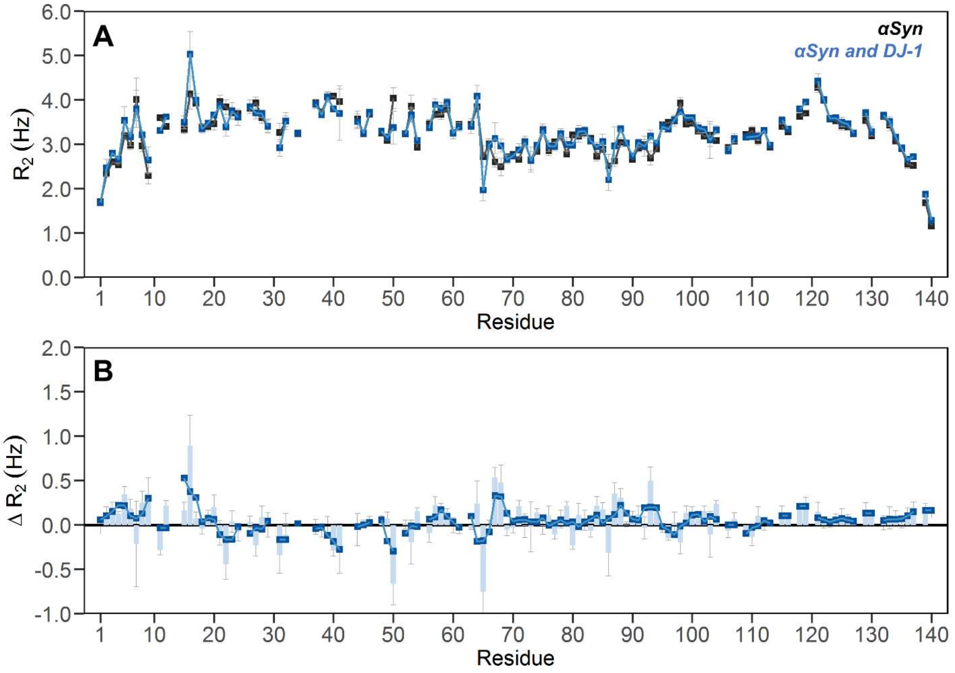

Figure S8. Impact of DJ-1 on ac-αSyn monomers. (A) Per-residue ^{15}N-R_2 values of ^{15}N-native ac-αSyn (250 μM) in the absence (black) or presence of 500 μM DJ-1 (blue). (B) Per-residue ΔR_2 values determined by subtracting the ^{15}N-R_2 values of native ac-αSyn in the absence of DJ-1 from those in the presence of DJ-1 (data plotted in A). Connected points in darker blue represent a three-point average and are drawn to guide the eye. The minimal changes in ^{15}N-R_2 for the ac-αSyn monomer suggest that the ac-αSyn monomer does not have a detectable interaction with DJ-1. ^{15}N-R_2 measurements were acquired at 700 MHz ^{1}H Larmor frequency at 15°C in PBS, pH 7.4.
